# Recommendations from Jane Gitschier's Bookshelf

**DOI:** 10.1371/journal.pgen.1004009

**Published:** 2013-12-05

**Authors:** Jane Gitschier

**Affiliations:** Institute for Human Genetics and Departments of Medicine and Pediatrics, University of California, San Francisco, San Francisco, California, United States of America

**About the Author**

Jane Gitschier is a human geneticist and Professor Emeritus at the University of California, San Francisco. She has served as the Interviews Editor for *PLOS Genetics* since its inception in 2005 and in that capacity has published 35 interviews of geneticists and others whose work dovetails with genetics. Jane has run a genetics book club for the past 10 years or so, and shares here a selection of her favorite reads.

Today *PLOS Genetics* launches a new frontmatter feature, the “Deep Reads” column, celebrating books in the realm of genetics and, in today's instance, beyond (Image 1). Kudos to our editorial colleague Susan Rosenberg for proposing the column's clever name. While I'm the kickoff contributor, various members of the *PLOS Genetics* community will pen subsequent columns, hopefully annually.

**Figure:**
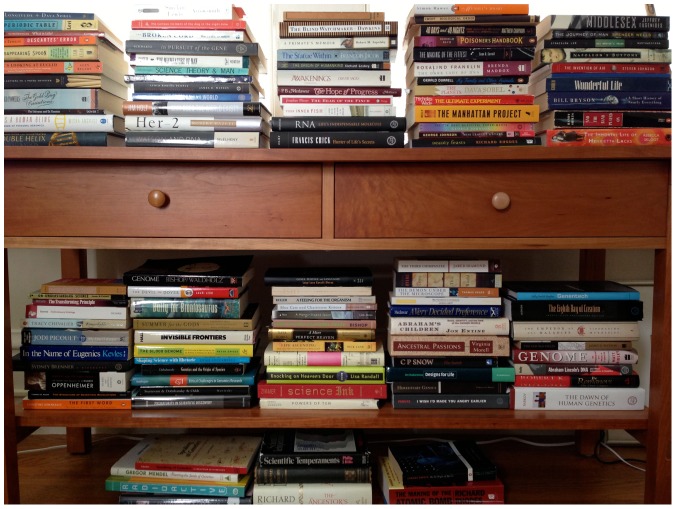
Jane Gitschier's bookshelf. Photo courtesy of Jane Gitschier.

Over the past decade I've had the pleasure of running a genetics book club, first as an occasional focus for my laboratory's group meeting, and later for the Institute of Human Genetics at UCSF (University of California, San Francisco). Many books have been suggested, digested, and discussed by these two enthusiastic sets of readers. We have cast a wide net, from genetics, human-inherited disease, and evolution, to chemistry, physics, and invention; we have also ranged from scientific narrative and biography to fiction and science fiction. I would like to acknowledge my fellow book lovers, as they have opened up new worlds of reading for me and have endured some of my own passions. Here, follow some of my personal top picks in this spectrum of books; I have chosen not to restrict my recommendations to genetics, as I suspect you too may enjoy reading about many aspects of science. My offering is not comprised of “deep” reviews in that my comments are neither very detailed nor highly critical; rather, it is a summing up, with brief description, of the books I've most enjoyed and highly recommend to you or your family and friends.

## Memoir

I begin with the memoir, my favorite genre—perhaps not surprisingly since I write the ;Interviews column for this journal. In this category, my hands-down top recommendation is James Watson's enduring ***The Double Helix***. If you haven't yet tagged along with this irreverent romp through the discovery of the structure of DNA, I urge you to do so. It is short and impossible to put down once you begin. The book generated quite a bit of controversy when it was written in the late 1960s; some participants in the drama argued that they were poorly portrayed and many others complained of its dismissive posthumous treatment of Rosalind Franklin. In truth, Watson's memoir doesn't portray Watson himself in any shining light either, and this is one of its charms. Watson's naked memoir captures an extraordinary two-year period when post-war Cambridge, England redirected its scientific energies towards solving fundamental biological problems, and a youthful Watson hijacked Francis Crick into thinking about DNA's structure. I recommend the new, annotated version, issued by Cold Spring Harbor Press in honor of the structure's 60^th^ birthday, which includes photos and documents that further bring the story to life, while also providing the historical tether that it is often accused of lacking.

***Uncle Tungsten*** is a memoir by the neurologist and incomparable storyteller Oliver Sacks. Sacks describes growing up in London circa WWII in a lively, large, and highly intellectual family, including an uncle—Uncle Tungsten—who runs a light bulb factory. Indeed, Tungsten isn't the only maternal uncle with a chemical bent; seven other maternal uncles worked in the field of mineralogy! The memoir is a homage to chemistry, with the feel of elements and the smell of experiments swirling in a Proustian reverie of a time when well-to-do families could afford to have chemistry laboratories in their own homes.

Another endearing memoir of boyhood is ***My Family and Other Animals***, in which the British naturalist and conservationist Gerald Durrell recounts his family's move from the rainy UK to sunny Corfu during the 1930s. There, the 10-year-old Durrell takes on the natural history of the island, securing a mentor who meets with him weekly to study the fauna he encounters and then bringing home terrapins and tortoises, birds and scorpions, indeed all manner of creatures to his tenderly and delightfully drawn family. Would that all of us could have had such an unfettered, exploratory childhood!

I also recommend a pseudo-memoir, ***The Search***, a first-person fictional account of one man's path in becoming a scientist. Writing in the early 1930s, C. P. Snow, himself a scientist-turned-author (who later gave the influential Rede Lecture in 1959 on the lack of communication between the arts and sciences), chronicles the intellectual, professional, and moral journey of his protagonist, Arthur Miles, a mirror for Snow himself. We meet Miles in the UK before WWI when he and his father look out at the night sky and young Arthur vows to become a scientist. He makes the decision to pursue chemistry, inspired by his high school teacher's enthusiasm for Niels Bohr and the newly described structure of the atom. Miles chooses the new field of X-ray crystallography for his life's work, first studying manganates, then later an unspecified biological problem. He plots out his career, jockeying to launch and lead a new institute and, ultimately, moving on from science altogether. I recommend this book because, even though the action takes place nearly a century ago, Snow, in the voice of Miles, eerily captures a passion, decision, discouragement, or dilemma that I myself have faced and probably you have, too. Graduate students take note: Miles's dearest lifelong friends are those he made in graduate school, and I think this will resonate for many readers.

## Biography

Sadly, few scientists take the time to chronicle their own experiences, so it is often left to others to piece them together. Here, I recommend four “biographies” (the quotes will become apparent in a minute), and as it happens, all of them are of women and written by women (just as the four recommended memoirs were by men). ***Rosalind Franklin: The Dark Lady of DNA***, by Brenda Maddox, patiently takes us through Franklin's careful crystallographic work on coal and her happy existence in Paris before repatriating to London with Randall's group to work on DNA; it then follows her highly productive post-DNA work with Aaron Klug on the structure of RNA viruses until her premature death. We are given the context to understand how Franklin came to work on the problem of DNA, the basis for the antipathy between her and Wilkins, her resiliency post-DNA, and her tenacity throughout isolation and disease. She likely died unaware of how critical the contribution of photograph 51, taken by her student Raymond Gosling, was to one of the 20^th^ century's great discoveries. By the way, be on the lookout for a play entitled ***Photograph 51***, by Anna Zeigler, which deftly covers this riveting story.

While Franklin famously eschewed model-building for hard facts, Barbara McClintock was the master of intuition. One of the most purposeful scientists of the 20^th^ century, McClintock is captured in Evelyn Fox Keller's biography ***A Feeling for the Organism***, published in 1983, coincidentally the year McClintock was awarded the Nobel Prize for her discovery of transposition. Though short, the book is not as easy a read as Maddox's biography of Franklin, in large part because of the difference in subject matter: we “get” the structure of DNA—it's iconic—but McClintock's work on maize's “controlling elements,” which can change position in chromosomes, baffled her contemporaries. Her publications were (and still are) nearly impossible to read, so much so that by the 1950s she simply ceased to publish her work altogether. Yet this book, based in large part on interviews with McClintock and her colleagues, is captivating. I particularly enjoyed learning about McClintock's training and early career in the maize group at Cornell, when she made significant cytogenetic discoveries such as the crossing-over during meiosis that supported chromosomal exchange as the physical basis for genetic recombination.

When I was a girl, there was no more prominent example of a female scientist than Marie Curie—this may still be the case. Unlike Franklin or McClintock, Curie coupled her extraordinary scientific life with a husband and children. In ***Radioactive: A Tale of Love and Fallout***, Lauren Redniss dazzles us with her departure from the typical biography: here imaginative presentation replaces the typical dreary march of chronology. This is a magical book, with luminous cyanotype artwork and even a typeface newly created by Redniss. Chapter titles have double meanings—e.g. “Magnetism” for the chapter when she meets her husband Pierre, as they both worked on magnetic properties at the time, and “Exposure” for the chapter on her revealed love affair with Paul Langevin, Pierre's student, following Pierre's death. Clearly written, the book succinctly follows the path through fin-de-siècle physics, but often interleaves scrapbook-like references to future implications of the Curies' discoveries (for example, Chernobyl and Hiroshima) as well as odes to Poland and poignant excerpts from Marie's diaries and letters.

My final entry in the “biography” category is technically historical fiction. The popular book ***Remarkable Creatures***, by Tracy Chevalier, envisions the life of fossil-hunter Mary Anning, who combed the shoreline of her native Lyme Regis and uncovered fossils of large, extinct marine reptiles, the ichthyosaurs and plesiosaurs. Anning's discoveries revolutionized thinking in the early 19^th^ century about the age of the earth and proved that creatures could become extinct, as radical a concept at that time as the evolution of new species. This story is so fascinating that it prompted one of our book club members to make a pilgrimage to Lyme Regis while visiting the UK. Indeed, even a visit to London's Natural History Museum will bring you up close and personal with Mary Anning and some of these fossils.

## Fiction

My book club and I are always on the lookout for works of fiction in which some aspect of genetics (or science, more generally) drives the plot. Unfortunately, many of these reads are unsatisfying to me; some are poorly researched or simply implausible, others are clearly fictionalized versions of actual events, yet pale in comparison to their real life stories. I must also sheepishly confess that I am not a fan of the “science fiction” genre; I know I am in the minority in this aversion, and surely other PLOS editors will extol their favorite sci-fi reads in future columns.

Occasionally, however, a novelist develops a character who suffers from a disorder which springs from a genetic or epigenetic perturbation, and from these challenges, a plot emerges. For me, as a human geneticist, some of these portrayals can be vivid, and here I recommend three examples. First is ***The Curious Incident of the Dog in the Night Time***, by Mark Haddon, who conveys the terror and determination of an autistic teenager struggling to find the murderer of a neighbor's dog. Heads up for those of you in or near London: do not miss the National Theatre's clever and moving production of a play based on this book. Second is ***Mendel's Dwarf***, by Simon Mawer, who tells two parallel stories: one is of the monk Gregor Mendel and his peas (we are right there in his cloistered garden with him), the other is of Mendel's distant relative, a scientist who searches for the genetic explanation for his hereditary dwarfism known as achondroplasia. Third is Jeffrey Eugenides' ***Middlesex***, an epic tale of Greek immigrants, the tumultuous Detroit in the '60s, and finally the genetic misfortune of Cal, born as a “girl,” but who upon adolescence discovers himself to be male, with a 46XY disorder of sexual development. Eugenides, whether he was conscious of it or not, throws out a red herring early in the plot, in which Cal's grandparents are actually brother and sister, whereas from a genetic point of view, it would have been more logical to have his parents as the first degree relatives. I forgave him this confusion because the book is so good. It is worth mentioning that one of the main characters in Eugenides' latest work, ***The Marriage Plot***, suffers from bipolar disorder, and this depiction, too, is compelling. Our book club read the latter because it has a Woods Hole/Cold Spring Harbor sub-plot, complete with yeast genetics and a Barbara McClintock-esque character, but these brief threads, to our disappointment, went nowhere.

## Nonfiction

And now for a quick dip into a science nonfiction grab bag of delights. In ***Napoleon's Buttons***, co-written by Penny Le Couteur and Jay Burreson, history meets chemistry as we learn how sugar, caffeine, dyes, tin, and a variety of other molecules shaped the course of human endeavor. Chemicals are also front and center in ***The Poisoner's Handbook***, an engaging inspection of murders and accidental deaths in prohibition-era New York City and the emergence of the forensic science needed to pinpoint the culprits. I thoroughly enjoyed ***Your Inner Fish***, by Neil Shubin, who illustrates how the seeming illogic of human anatomy reveals the vestiges of evolution. If paleoanthropology interests you—and how can it not—look for ***Ancestral Passions***, by Virginia Morell, who traces the indefatigable Leakey family in their multi-generational search for human ancestors; I am not a “night” person, but this tale had me turning pages way past midnight several nights in a row, and Olduvai Gorge is now on my bucket list. In the brilliant ***The Emperor of All Maladies***, Siddhartha Mukherjee takes us through the history of cancer awareness and its treatment; the descriptions of early breast cancer surgeries are particularly difficult to contemplate and the work of Sidney Farber was thrilling to read. ***And the Band Played On***, by Randy Shilts, who was a journalist with the *San Francisco Chronicle*, is an unrelenting exposé on both the political mayhem and the dogged quest to solve an urgent medical mystery at the emergence of the AIDS epidemic.

But wait, there is more! In his tour de force ***The Eighth Day of Creation***, Horace Freeland Judson chronicles two decades that form the dawn of molecular biology, and his extensive interviews allow us to hear the participants' voices; I was most intrigued by the section centered on the Institut Pasteur, in which a small number of gripping and intimately connected individuals started with very simple questions about bacteriophage biology and sugar metabolism and ended up discovering gene regulation and the operon. Stephen Hall picks up the pulse of molecular biology in the late 1970s in ***Invisible Frontiers***, a fast-paced account of the bicoastal race to clone the human insulin gene at the birth of the biotechnology industry amid the recombinant DNA moratorium; this was a particularly fun read for me, as I happened to know many of the participants in the story, but I think that anyone with an interest in that pivotal technology would enjoy it. ***Miss Leavitt's Stars***, by George Johnson, is a delightful and illuminating story about the cosmos; it is part biography and part explanation of how Henrietta Leavitt, one of a cluster of female “human computers” who calculated star brightness from large photographic plates at the turn of the 20^th^ century, discovered a relationship between the brightness and the periodicity of “variable” stars and correctly interpreted that their absolute luminosity could then be used as a standard candle to measure the distance to other stars. Dava Sobel's ***Longitude*** tells the 18^th^ century tale of the exasperating competition to accurately calculate longitude at sea; I found the story of Harrison and his exquisite clocks so interesting that I had to see them at the Greenwich Royal Observatory. Finally, I bow to Richard Rhodes, author of my all-time favorite science narrative ***The Making of the Atomic Bomb***. Do not be intimidated by a little nuclear physics! This book is a lucid page-turner: the story is both magnificent, speaking to the genius and industry of men and women working under the incredible pressure of war, as well as terrifying in its implications, and we feel the tension in it.

I close with a teaser for a great genetics read, ***In Pursuit of the Gene***, and shameless promotion for my next interview, to be published in early 2014, with its author James Schwartz. Go out and grab a copy, and until then, keep those pages turning!

